# A review of tangle-veined flies (Nemestrinidae, Diptera) in Egypt

**DOI:** 10.3897/zookeys.1071.70743

**Published:** 2021-11-16

**Authors:** Arafa Elsayed El-Hashash, Haitham Badrawy Mousa Badrawy, Ayman Mohyie-Eldin Ibrahim

**Affiliations:** 1 Department of Taxonomy, Plant Protection Research Institute, Agricultural Research Centre, Dokki-Giza, Egypt Department of Taxonomy, Plant Protection Research Institute Dokki-Giza Egypt; 2 Plant Protection Department, Agricultural Faculty, Peoples’ Friendship University of Russia, Moscow, Russia Peoples’ Friendship University Moscow Russia; 3 Department of Entomology, Faculty of Science, Ain Shams University, Abbassia-Cairo, Egypt Ain Shams University Abbassia-Cairo Egypt

**Keywords:** Distribution, Egypt, Nemestrinus, taxonomy

## Abstract

The Egyptian fauna of the genus *Nemestrinus* Latreille, 1802 is revised. In 1967, Steyskal and El-Bialy listed 12 species from the region, but only six species are now recognized. The primary type specimens of the species *N.aegyptiacus* (Wiedemann, 1828), *N.rufipes* (Olivier, 1810), and *N.lateralis* Wiedemann, 1828 (*N.lateralis* being a synonym of *N.rufipes*) deposited in the Museum für Naturkunde, Berlin, Germany were examined. Two species (*N.abdominalis* Olivier, 1811 and *N.fascifrons* (Bigot, 1888) are placed as new synonyms of *N.ater* (Olivier, 1811), and *N.ruficornis* Macquart, 1840 is synonymized with *N.rufipes* (Olivier, 1811). *Nemestrinusjullieni* (Efflatoun, 1925) is confirmed as a synonym of *N.aegyptiacus*. Furthermore, three species (*N.caucasicus* Fischer, 1806, *N.pallipes* (Olivier, 1811), and *N.persicus* Lichtwardt, 1909) have been removed and are doubtful records from Egypt. A key to the species, lists of specimens examined, and Illustrations and distributions for each species are provided. The status of species of doubtful occurrence in Egypt is discussed.

## Introduction

Nemestrinidae (tangle-veined flies) are a small dipteran family belonging to the suborder Brachycera-Orthorrhapha and occur all over the world, but are most abundant and diverse in the Palaearctic, Australian and Afrotropical Regions (Richter 1997; Pape et al. 2011). The family is classified into five subfamilies and comprises approximately 300 species worldwide in 23 genera, while 77 species in eight genera are known from the Palaearctic region (Richter 1997; Narchuk 2007; Papavero and Bernardi 2009; Pape et al. 2011).

The Nemestrininae comprise *ca.* 175 species worldwide in six genera (Bernardi 1973; Papavero and Bernardi 2009). In the Palaearctic Region, the Nemestrininae currently include approximately 67 species in two genera (*Nemestrinus* Latreille, and *Stenopteromyia* Lichtwardt) according to the last published catalogue by Richter (1988).

The genus *Nemestrinus* was described by Latreille in 1802 based on specimens collected from Egypt and Syria. It comprises 66 species in the Palaearctic Region (Bernardi 1973; Richter 1988, 1997; Narchuk 2007) and is characterized by the wing venation: The apical part of the wing occasionally has supernumerary transverse veins, R_3_ is present, R_3+4_ and R_5_ are free, M_1_ and M_2_ are free, the diagonal vein reaches the wing margin, and the proboscis is well developed and longer than the head. One of the important tools to separate nemestrinid species is the genitalia, composed of the well-developed hypandrium, partly fused with the gonocoxites with a linguiform apical projection bearing numerous hairs; and the elongate gonocoxal apodemes, which are sinuate and fused medially forming a dorsal bridge (Richter and Ovtshinnikova 1996; Richter 1997).

*Nemestrinus* is primarily distributed along the arid desert belt of the Palaearctic Region where several species occur in North Africa (Morocco, Algeria, Tunisia, Libya, and Egypt) and the Middle East (Arabia, Israel, Iran), east to Central Asia, as far as Mongolia and southern Russia, and in addition southern Europe (Bulgaria, Romania, Ukraine, France, Spain, and Turkey). The genus penetrates south into the Saharan part of the Afrotropical Region, being recorded from Sudan and Ethiopia (Bernardi 1973; Richter 1988, 1997). The type localities of seven species are situated in Egypt: *N.abdominalis* Olivier, 1811, *N.aegyptiacus* (Wiedemann, 1828), *N.ater* (Olivier, 1811), *N.fasciatus* (Olivier, 1811), *N.reticulatus* Latreille, 1802, *N.ruficornis* Macquart, 1840, and *N.rufipes* (Steyskal and El-Bialy 1967; Bernardi 1973; Richter 1988).

Two catalogues cover the nemestrinid fauna of Egypt: the monograph of Sack (1933) lists ten species and one variety [*Nemestrellusabdominalis*, *N.ater*, *N.exalbidus* (Lichtwardt, 1907), *N.fascifrons*, *N.ruficornis*, *N.rufipes*, *Nemestrinusaegyptiacus*, *Ne.a. var. jullieni*, *Ne.persicus*, *Ne.reticulatus*, and *Rhynchocephalusfasciatus*] in three genera (*Nemestrellus*, *Nemestrinus*, *Rhynchocephalus*), while Steyskal and El-Bialy (1967) list eleven species and one variety in the same three genera, adding *R.caucasicus* to Sack’s monograph. There are also outdated works (e.g., Bequaert 1932, 1938) on the taxonomic status of the genus. Bernardi (1973) reviewed the world genera, and Richter (1988) presented the Palaearctic catalogue of Nemestrinidae; both listed ten species in Egypt, removing the same two species (*Nemestrinuscaucasicus* and *N.persicus*) listed by Steyskal and El-Bialy (1967). Bernardi (1973) and Richter (1988) added *N.pallipes* that was not listed in Steyskal and El-Bialy (1967) as Egyptian species.

There is no modern comprehensive work identifying and cataloguing the Egyptian nemestrinine fauna. The subfamily in Egypt has never been monographed, and the genus is very much in need of a modern revision. This study was undertaken to revise, update, and clarify the taxonomic status of the species of genus *Nemestrinus* Latreille in the Egyptian fauna.

## Materials and methods

Specimens examined in this study are deposited in the following collections:

**ASUC** Entomology Department, Faculty of Science, Ain Shams University

**AZUC** Faculty of Agriculture, Alfieri, Al Azhar University

**CUC** Entomology Department, Faculty of Science, Cairo University

**ESEC** Entomological Society of Egypt

**MAC** Department of Taxonomy, Plant Protection Institute, Ministry of Agriculture

**NHMW** Naturhistorisches Museum Wien, Austria

The Museum für Naturkunde, Germany, Berlin (**ZMHB**) is the depository of type specimens of *N.aegyptiacus*, *N.rufipes*, and *N.lateralis* Wiedemann, 1828 (the latter is a synonym of the second species). We obtained this information by personal communication with Mr. Sven Marotzke and Ms. Elena Grigoryeva.

We could not access the types of other species because some are missing, as in the Egyptian Society of Entomology, wherein type specimens of the species *N.jullieni* have apparently been destroyed, and it is not known where the other types are. We examined and revised the original descriptions of all Egyptian nemestrinid species.

The Smithsonian National Museum of Natural History (**USNM**) has specimens of Nemestrinidae from Egypt: *N.abdominalis*, *N.aegyptiacus*, and *N.rufipes* that have all been identified by Dr. Torsten Dikow using our key. Redescriptions are based on series of specimens of each of these species and body measurements include genitalia.

Morphological terms follow McAlpine et al. (1981), Richter and Ovtshinnikova (1996), Richter (1997) and Cumming and Wood (2017). Line drawings of body parts were made by using a stereomicroscope at a magnification of 40×. We have access to the photographs by Ms. Elena Grigoryeva, Mr. Sven Marotzke, and Bernhard Schurian of the types that were downloaded at https://doi.org/10.7479/4wgc-dv22.

## Taxonomic account

### Nemestrinus

Taxon classificationAnimaliaDipteraNemestrinidae

694F9D03-4D8D-55D9-9F49-3B9453F61225

Nemestrinus Latreille, 1802: 437. Type species: Nemestrinusreticulatus Latreille, 1802: 437.
Rhynchocephalus Fischer, 1806: 219–220.
Andrenomyia Rondani, 1850: 189.
Heminemestrinus Bequaert, 1932: 21.
Symmictoides Bequaert, 1932: 105.
Nemestrellus Sack, 1933: 7.
Nemestrina Rondani, 1850: 189, 197: incorrect subsequent spelling of Nemestrinus Latreille, 1802 or subsequent usage of Nemestrina Blanchard, 1845: 468.

#### Remarks.

 Currently there are six species the Egyptian fauna (*N.aegyptiacus*, *N.ater*, *N.exalbidus*, *N.fasciatus*, N. *reticulatus*, and *N.rufipes*). The type specimens of *N.jullieni* deposited in ESEC have been destroyed by dermestid beetles and the types of the species *N.aegyptiacus* and *N.rufipes* and the type of latter’s synonym *N.lateralis* are deposited in ZMHB.

Three species (*N.caucasicusN.pallipes*, and *N.persicus*) have been treated as doubtful since there is no evidence of their occurrence in Egypt. This is based on their known distributions as listed in the world catalogue by Bernardi (1973), the Palaearctic catalogue by Richter (1988), and the Systema Dipterorum (Thompson and Pape 2021). Additionally, the type localities of *N.caucasicus* and *N.persicus* are in the Caucasus, Iran, and Jaffa (Israel) respectively, not in Egypt.

### Key to the Egyptian species of Nemestrinus

**Table:** 

1	Wing without supernumerary transverse veins (Fig. 65)	***N.fasciatus***
–	Wing with supernumerary transverse veins, resulting in reticulate venation (Fig. 8)	**2**
2	Small cells extending forward posterior to R1 (Fig. 27); frons shiny black with a transverse white band	***N.ater***
–	Small cells extending forward posterior to R2 (Fig. 18); frons entirely pollinose or with a shiny spot	**3**
3	Small cells restricted between R2 and hind margin (Fig. 85); abdomen entirely black or grey with transverse black stripes	**4**
–	Small cells restricted between R2 and M1 or M2 (Figs 46, 90); abdomen orange with a longitudinal black vitta (Figs 57, 101)	**5**
4	Abdomen entirely black (Fig. 1); frons entirely pollinose (Figs 4, 5)	***N.aegyptiacus***
–	Abdomen gray with incomplete transverse black stripes (Fig. 84); frons with a shiny spot (Figs 80, 81)	***N.reticulatus***
5	Frons yellowish black; vertex black; venter of abdomen black with yellowish incisions	***N.pallipes***
–	Frons yellow or grey; vertex black or brown; venter of abdomen entirely orange or with black sides	**6**
6	Frons yellow pollinose; tergum II with a transverse white band (Figs 2, 3, 91); venter of abdomen orange and black laterally	***N.rufipes***
–	Frons grey pollinose; tergum II without a transverse white band (Fig. 47); venter of abdomen entirely orange	***N.exalbidus***

### Nemestrinus aegyptiacus

Taxon classificationAnimaliaDipteraNemestrinidae

Wiedemann, 1828

0EEB72E0-CDFF-5F6C-9304-805465207152

Figures 1 , 4–13 , 14–22

Nemestrinus aegyptiacus Wiedemann, 1828: 249.
Nemestrinus tripolitana Lichtwardt, 1907: 443.
Nemestrinus jullieni Efflatoun, 1925: 357.

#### Type material.

*Nemestrinusaegyptiacus*: Syntype female, without date, Egypt (ZMHB) (pers. comm., Mr. Sven Marotzke). *Nemestrinusjullieni*: Type W. Hoff 29°53'02.6"N, 31°18'42.2"E , 15.iii.1922, Helwan 29°50'37.6"N, 31°19'05.0"E , 20.iii.1925; Lectotype male “W. Hoff 29°53'02.6"N 31°18'42.2"E, 23.iii.1922”, Egypt (formerly ESEC, destroyed by dermestid beetles).

#### Specimens examined.

*N.aegyptiacus*: Burg El-Arab 30°54'12.7"N, 29°33'13.7"E, 25.iii.1927 (1 f#), 25.iii.1934 (1 m#); Helwan 29°50'37.6"N, 31°19'05.0"E, 17.iii.1934 (1 f#), 16.iii.1935 (1 f#); W. dar El Maskhara 29°47'02.9"N, 31°24'59.9"E , 11.iv.1927 (1 f#); W. Garawi 29°47'43.9"N 31°25'54.9"E, 22.iii.1930 (1 f#), 31.iii.1930 (1 m#); W. Hoff 29°53'02.6"N, 31°18'42.2"E, 10.iii.1930 (2 m#) (AZUC); Abu Rawash 30°04'30.7"N, 31°11'59.7"E, 7.iii.1955 (9 m#), 8.iii.1955 (3 m# & 1 f#), 13.iii.1955 (4 m# & 3 f#), 17.iii.1955 (5 m# & 4 f#), 20.iii.1955 (2 m# & 5 f#); Giza 30°00'40.0"N, 31°11'31.4"E, 22.iii.1954 (1 m#), 17.v.1955 (1 m#); Helwan 29°50'37.6"N, 31°19'05.0"E, 17.iii.1934 (1 m#), 20.iii.1934 (1 m#), 3.iv.1934 (1 f#); Ogret El-Sheik 29°52'50.1"N, 31°18'27.8"E , 25.ii.1927 (1 m#); W. Garawi 29°47'43.9"N 31°25'54.9"E, 25.iii.1932 (1 f#); W. Rishrash 29°27'51"N, 31°22'2"E, 29.iii.1935 (1 f#); W. Silly Helwan 29°50'37.6"N, 31°19'05.0"E, 19.iii.1926 (1 f#); Ain Mousa 29°52'22.0"N, 32°39'00.7"E , 16.iii.1925 (2 f#) (CUC); Asyut (Lentil) 27°23'00.0"N, 31°44'38.0"E, 3.iii.1965 (2 m# & 6 f#); Ogret El-Sheik 29°52'50.1"N, 31°18'27.8"E, 21.iii.1926 (2 f#); Burg El-arab 30°54'12.7"N, 29°33'13.7"E, 25.iii.1927 (1 m#), 9.iii.1928 (1 m#); Kafr Hakim 30°04'39.7"N, 31°06'46.3"E, 24.iii.1924 (1 m#); Gerga (Eg. Lupia) 26°20'23.2"N, 31°53'21.3"E , 2.iv.1965 (2 f#); W. Hoff 29°53'02.6"N, 31°18'42.2"E, 21.iii.1922 (1 f#), 22.iii.1927 (1 m#); W. Morrah 22°22'39.1"N, 33°46'00.3"E, 25.iii.1921 (1 m#); W. Silly Helwan 29°50'37.6"N, 31°19'05.0"E, 23.iii.1926 (1 m#), 25.iii.1927 (2 f#) (MAC); Abu Mena 30°50'28"N, 29°39'49"E, 15.iii.1953 (1 m#), 8.iv.1954 (3 m#); Gabal Asfar 30°12'05.7"N 31°21'19.7"E, 9.iii.1951 (1 f#), 19.iii.1951 (1 f#); Kerdasa 30°01'32.1"N, 31°06'27.5"E, 14.iv.1951 (1 f#), 20.iii.1952 (1 f#) (ASUC); (1 m#), without data (NHMW) sent by Dr. Peter Sehnal; Egypt (1 f#), without date, (ZMHB) sent by Mr. Sven Marotzke and Bernhard Schurian; Cairo, Shoubra, 30°4'27.1632"N, 31°14'53.9844"E, 28.iii.1921 (1 m#), specimen number USNMENT01371555 (USNM) (identified by Dr. Torsten Dikow).

Specimens previously identified as *N.julieni*: Abu Rawash 30°04'30.7"N, 31°11'59.7"E, 7.iii.1955 (1 m#), 8.iii.1955 (1 m#), 13.iii.1955(1 m#); Burg El-Arab 30°54'12.7"N, 29°33'13.7"E, 25.iii.1934 (4 m# & 4 f#); W. Garawi 29°47'43.9"N, 31°25'54.9"E, 25.iii.1932 (2 f#), 21.iii.1930 (1 f#); W. Hoff 29°53'02.6"N, 31°18'42.2"E, 28.ii.1927 (1 m#); W. Um Elek 29°52'59.9"N, 31°31'00.1"E, 21.iii.1924 (1 f#) (CUC); Ain Mousa 29°52'22.0"N, 32°39'00.7"E , 16.iii.1925 (2 f#); Asyut (Lentil) 27°23'00.0"N, 31°44'38.0"E, 3.iii.1965 (1 m# & 2 f#); Burg El-Arab 30°54'12.7"N, 29°33'13.7"E, 14.iv.1920 (1 m#), 16.ii.1922 (1 f#),12.iv.1923 (1 f#),11.iv.1925 (1 m#), 18.iv.1925 (1 f#); Kafr Hakim 30°04'39.7"N, 31°06'46.3"E, 20.iv.1925 (1 f#); W. Garawi 29°47'43.9"N, 31°25'54.9"E, 14.iv.1928 (1 f#); W. Morrah 22°22'39.1"N, 33°46'00.3"E, 25.iii.1927 (1 f#), W. Silly Helwan 29°50'37.6"N, 31°19'05.0"E, 19.iii.1926 (2 f#), 22.iii.1926 (1 f#), W. Um Elek 29°52'59.9’’ N, 31°31'00.1"E, 21.iii.1924 (1 f#) (MAC); Kosseir 26°06'26.2"N, 34°16'38.8"E, 24.ii.1965 (1 m#); W. Digla 29°59'00.1"N, 31°19'41.2"E, 5.iv.1952 (1 f#), 13.iii.1955 (1 m#); W. Natroun 30°25'58.2"N, 30°14'39.2"E, 13.iii.1955 (3 f# & 1 m#), (2 m# & 1 f#), without data (ASUC).

#### Diagnosis.

 Frons and face entirely yellow or grayish pollinosity; thorax completely shiny black with yellowish hairs; wing with many small cells restricted between R2 and hind margin; abdomen entirely black with short erect hairs. Male genitalia with only outer gonocoxal process; gonocoxal apodemes long, narrow, sinuate, fused medially and forming a narrow dorsal bridge; gonostyli wider than gonocoxal processes, ventrally with a cleft and small projection. Aedeagal complex with tapered aedeagus and lateral parameres, which are usually separated apically, fused with basal part of the aedeagus; parameral apodeme rather long; ejaculatory apodeme long and broad.

#### Redescription.

 Length: male body 14–17 mm, wing 13.5–15 mm. Female body 14–20 mm, wing 13.5–16.5 mm. Head wider than thorax; frons with yellow or grayish yellow pollinosity, with rather long hairs, at antennal elevation frons wide but narrowing toward vertex; face relatively shorter than high, with dull pollinosity, its hairs similar to those of the frons (Figs 4–6, 14–16); antenna entirely blackish brown to black, scape and pedicel with long hairs (Figs 7, 17). Thorax shiny black; mesonotum with dense pale yellow hairs but rather long; pleurae with tuft-like hairs. Leg hairy, yellowish brown with black femora. Wing smoky brown, except apex and posterapical margin clear and transparent (Figs 8, 18). The differences in cell number and structure on the wing is continuous variation and inconsistently different between males and females. Abdomen entirely shiny black, covered with yellowish pubescence except the venter is blackish, which is rather short and erect, excluding dorsal side of two basal segments and on lateral margins of second segment where it is much longer and tufted, also on hind margin of each segment appear as narrow light bands (Figs 9, 19). Male genitalia with only outer gonocoxal process; gonocoxal apodemes long, narrow, sinuate, fused medially, and forming a narrow dorsal bridge; gonostyli wider than gonocoxal processes, ventrally with a cleft and small projection (Figs 10, 11). Aedeagal complex with tapered aedeagus and lateral parameres, which are usually separated apically, fused with basal part of aedeagus; parameral apodeme rather long; ejaculatory apodeme long and broad (Figs 12, 13). Female genitalia with subgenital plate rectangular with two hairy lobes (Fig. 20); genital furca free, narrow, with broadened ends of posterior projections, bent medially; median aperture of genital furca nearly triangular (Fig. 21); uterus rounded, with two narrow and rather long spermathecae (Fig. 22).

#### Local distribution.

 Coastal strip, Lower Nile.

#### Geographical distribution.

 Algeria, Egypt, Libya, Morocco, Italy (Sicily), and Tunisia (Sack 1933; Bernardi 1973; Richter 1988).

#### Remarks.

**Figure 1.**
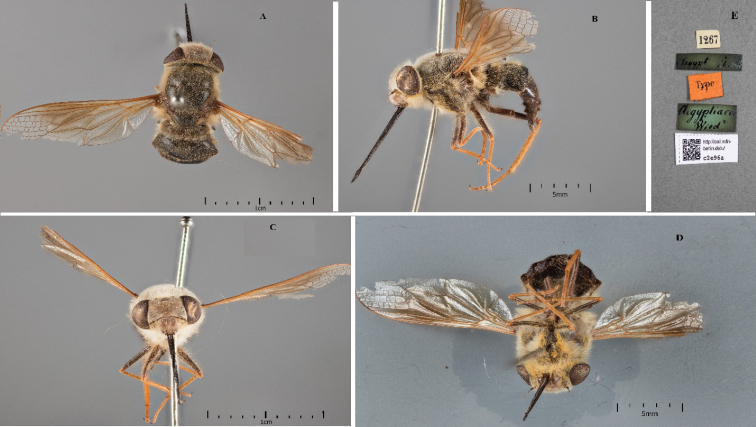
*Nemestrinusaegyptiacus*, female syntype **A** dorsal view **B** lateral view **C** frontal view **D** ventral view **E** labels (Zmhb).

 After examining the female type specimen of *Nemestrinusaegyptiacus* (Fig. 1) and comparing it with a large series of specimens identified as *Nemestrinusjullieni* Efflatoun (some specimens were by seen by him but it is not clear who determined the identification), we confirm this identification, and it is clear that both are the same species. Hence, *N.julieni* is placed as a synonym instead of a subspecies based on examination of the series of specimens and dissections of genitalia of both *N.aegyptiacus and N.julieni* and the female type specimen of *N.aegyptiacus*, and comparisons with the genitalia figures of Bernardi’s (1973: figs 54–56) *N.aegyptiacus*.

**Figure 2.**
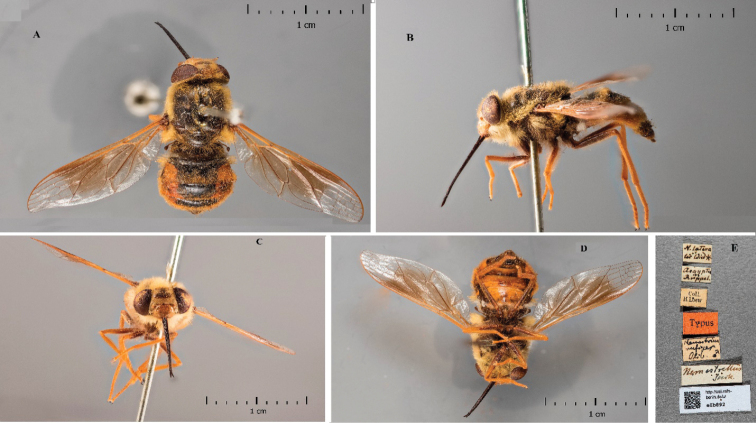
*Nemestrinusrufipes*, male type **A** dorsal view **B** lateral view **C** frontal view **D** ventral view **E** labels (Zmhb).

**Figure 3.**
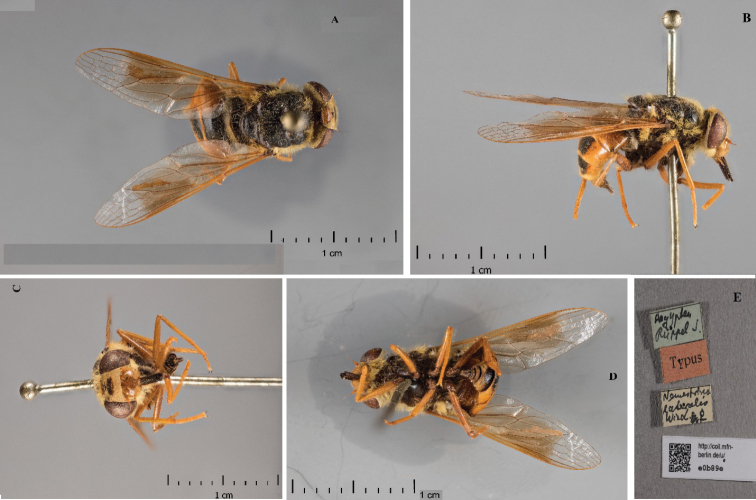
*Nemestrinuslateralis* (synonym), female syntype **A** dorsal view **B** lateral view **C** frontal view **D** Ventral View **E** Labels.

**Figures 4–13.**
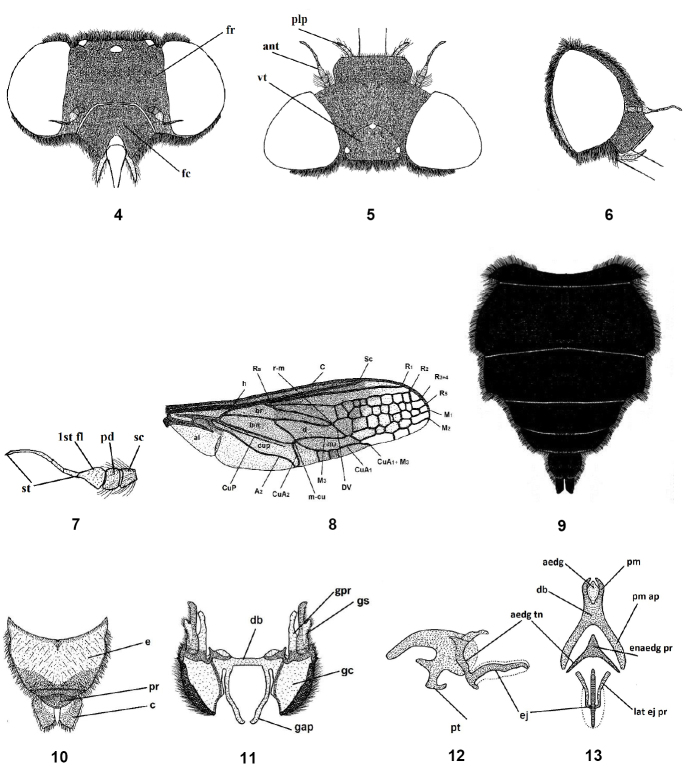
Male of *Nemestrinusaegyptiacus*, head, frontal (**4**), head, dorsal (**5**), head, lateral (**6**), antenna (**7**), wing (**8**), abdomen (**9**). **10–13** male genitalia: epandrium, proctiger, and cerci (**10**), gonocoxite with gonostylus, ventral (**11**), aedeagal complex, lateral (**12**) and dorsal (**13**). Abbreviations: aedg. aedeagus, aec. aedeagal complex, aedg tn. aedeagal tine A2. anal vein, al. alula, ant. Antenna, bm. basal medial cell, br. basal radial cell, c. cerci, C. costa, CuA1,2, CuP. cubital veins, d. discal cell, db. dorsal bridge, DV. diagonal vein, e. epandrium, ej. ejaculatory apodeme, enaedg pr. endoaedeagal process, fc. Face, 1st fl. first flagellomere, fr. Frons, gap. gonocoxal apodeme, gc. gonocoxite, gpr. gonocoxal process, gs. gonostylus, h. hypandrium, h. humeral cross vein, lat ej pr. lateral ejaculatory process, m3. third medial cell, pm. parameres, pm ap. parameral apodeme, pr. proctiger, pt. phallic plate, M1, M2, M3. medial veins, m-cu. cross vein between medial and cubital veins, pd, pedicel, plp. Palpus, R1, R2, R3+4, R5,Rs. radial veins, r-m. cross vein between redial and medial veins, Sc. subcostal vein, sc. Scape, st. stylus, vt. vertex.

**Figures 14–22.**
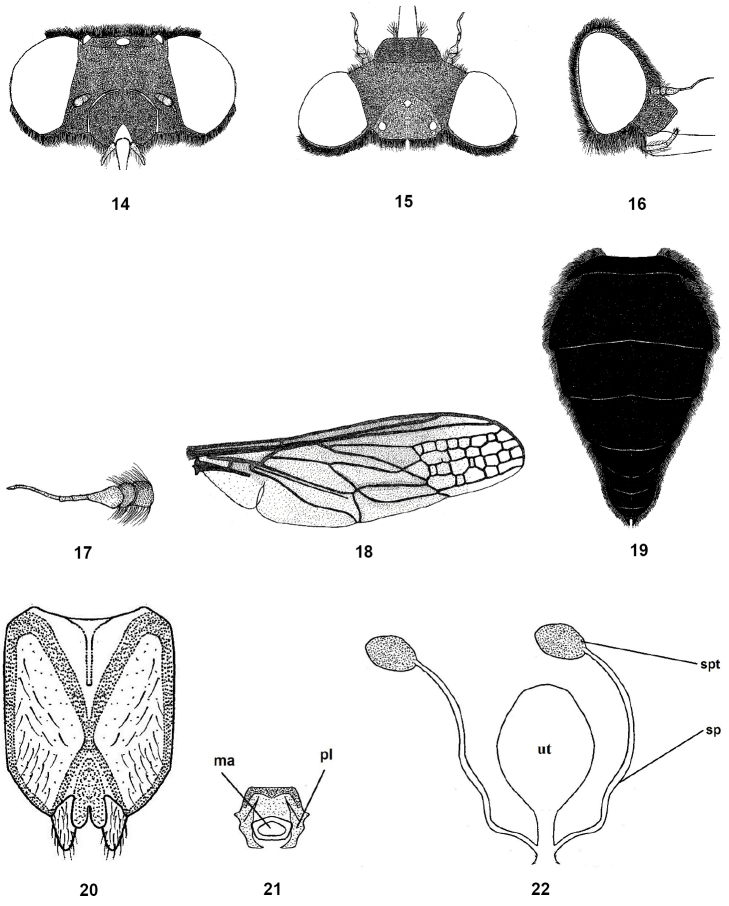
Female of *Nemestrinusaegyptiacus*, head, frontal (**14**), head, dorsal (**15**), head, lateral (**16**), antenna (**17**), wing (**18**), and abdomen (**19**). **20–22** female genitalia: subgenital plate (**20**), genital furca (**21**), and spermathecae (**22**). Abbreviations: ma. median aperture, pl. posterolateral projection, sp. spermatheca, spd. spermathecal duct, ut. uterus.

### Nemestrinus ater

Taxon classificationAnimaliaDipteraNemestrinidae

Olivier, 1811

17E3DAE8-58AD-5528-87EE-75237EB709EE

Figures 23–32 , 33–41

Nemestrinus ater Olivier, 1811: 171.
Nemestrinus abdominalis Olivier, 1811: 171. Syn. nov.
Nemestrinus nigra Wiedemann, 1828: 560.
Nemestrinus osiris Wiedemann, 1828: 561.
Nemestrina fascifrons Bigot, 1888: 8. Syn. nov.

#### Type locality.

Egypt.

#### Specimens examined.

 Abu Rowash 30°04'30.7"N, 31°11'59.7"E, 24.ii.1926 (1 f#), 26.ii.1927 (1 f#); Rafah 31°17'03.0"N, 34°14'18.0"E , 25.iv.1921 (1 f#); W. Garawi 29°47'43.9"N, 31°25'54.9"E, 31.iii.1930 (1 f#) (AZUC); W. Garawi 29°47'43.9"N, 31°25'54.9"E, 22.iii.1930 (1 f#), 31.iii.1930 (1 f#) (CUC); Abu Qir 31°18'42.4"N, 30°03'37.3"E, 26.iii.1915 (1 f#); (Noaman Bey) Alexandria 31°10'03.5"N, 29°51'56.2"E, (1 f#, without date); Dekheila 31°07'32.0"N, 29°48'37.3"E, 4.iii.1928 (1 f#); Dekheila Mariut 31°07'32.0"N, 29°48'37.3"E, 24.v.1925 (1 f#); Burg El-Arab 30°54'12.7"N, 29°33'13.7"E, 19.iv.1928 (1 f#); Gabal Abu Rowash 30°04'30.7"N, 31°11'59.7"E, 13.ii.1924 (1 f#); 19.iii.1924 (1 f#); Kafr Hakim 30°04'39.7"N, 31°06'46.3"E, 24.iii.1925 (1 f#), 20.iii.1926 (1 f#); Mansouriah 29°58'05.3"N, 31°08'51.9"E, 4.iii.1934 (2 f#); Burg El-Arab 30°54'12.7"N, 29°33'13.7"E, 18.iv.1925 (1 f#); Marsa Matrouh 31°11'04.1"N, 27°15'42.4"E, 17.iii.1933 (2 f#); Suize 29°58'09.6"N, 32°32'59.8"E, 5.iv.1927 (1 f#); Sinai N.E. 31°15'49.4"N, 34°10'15.8"E, 19.iv.1928 (1 f#); W. Silly Helwan 29°50'37.6"N, 31°19'05.0"E, 22.iii.1926 (1 f#) (MAC); Abu Rawash 30°04'30.7"N, 31°11'59.7"E, 26.iii.1952 (1 f#); Gabal Asfar 30°12'05.7"N, 31°21'19.7"E, 9.iii.1951 (1 f#); Mansoura 31°02'43.1"N, 31°22'54.9"E, 2.iii.1955 (1 f#); Mansouriah 29°58'05.3"N, 31°08'51.9"E, 12.iii.1952 (1 f#); Pyramids 30°04'39.8"N, 31°00'53.4"E, 12.iii.1951 (1 f#); W. Natroun 30°25'58.2"N, 30°14'39.2"E, 2.iv.1951 (1 f#) (ASUC).

Specimens previously identified as *N.fascifrons*: Abu Rowash 30°04'30.7"N, 31°11'59.7"E, 6.ii.1926 (2 m#); Helwan 29°50'37.6"N, 31°19'05.0"E, 18.iii.1927 (1 m#), 7.iii.1930 (1 m#); Mansouriah 29°58'05.3"N,31°08'51.9"E , 18.ii.1926 (1 m#); Mariut 31°08'32.5"N, 29°54'10.5"E , 5.iv.1921 (1 m#); W. Garawi 29°47'43.9"N, 31°25'54.9"E, 22.iii.1930 (1 m#), 31.iii.1930 (1 f#); W. Morrah 22°22'39.1"N, 33°46'00.3"E, 26.iii.1927 (1 m#) (AZUC); Kafr Hakim 30°04'39.7"N, 31°06'46.3"E, 20.iii.1926 (1 m#); Mansouriah 29°58'05.3"N, 31°08'51.9"E, 13.ii.1926 (1 m#), 2.iii.1927 (1 m#); W. Garawi 29°47'43.9"N, 31°25'54.9"E, 22.iii.1930 (1 m#), 31.iii.1930 (2 m#) (CUC); Dekheila 31°07'32.0"N, 29°48'37.3"E, 4.ii.1928 (2 m#), 4.iii.1928 (1 m#); Burg El-Arab 30°54'12.7"N, 29°33'13.7"E, 19.iv.1923 (1 m#); Kerdasa 30°01'32.1"N, 31°06'27.5"E, 15.ii.1923 (1 m#), 10.ii.1925 (1 m#); Mansouriah 29°58'05.3"N, 31°08'51.9"E, 13.ii.1926 (1 m#), 6.iii.1926 (1 m#); Burg El-Arab 30°54'12.7"N, 29°33'13.7"E, 27.iv.1923 (1 m#), 18.iv.1925 (5 m#); (Six Towers) Suize Road 29°59'45.9"N, 32°29'34.4"E , 26.iii.1926 (1 m#); Gabal Asfar 30°12'05.7"N, 31°21'19.7"E, 9.iii.1951 (1 m#); Mansoura 31°02'43.1"N, 31°22'54.9"E, 2.iii.1955 (1 m#) (ASUC).

Specimens previously identified as *N.abdominalis*: Egypt (1 f#), without date, specimen number USNMENT01371553 (USNM) (previously identified by W. Wirth as *N.abdominalis* but as *N.ater* by Dr. Torsten Dikow using our key).

#### Diagnosis.

 Frons shiny black with a transverse white band; wing with small cells extending forward from R1 to hind margin; abdomen orange with longitudinal black vitta in male but entirely black in female.

#### Redescription.

**Figures 23–32.**
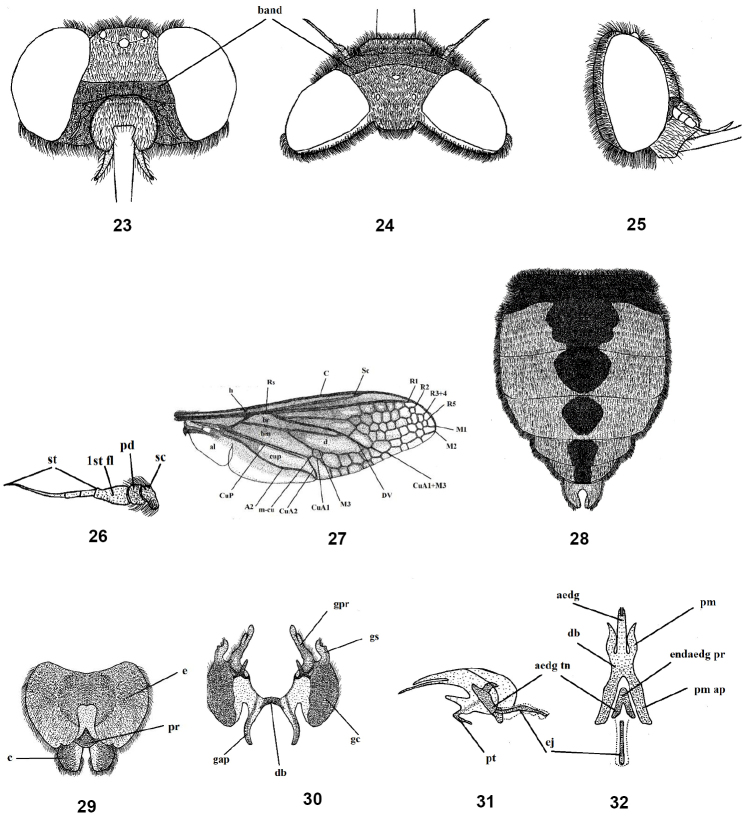
Male of *Nemestrinusater*, head, frontal (**23**), head, dorsal (**24**), head, lateral (**25**), antenna (**26**), wing (**27**), and abdomen (**28**). **29–32** male genitalia: epandrium, proctiger, and cerci (**29**), gonocoxite with gonostylus, ventral (**30**), aedeagal complex, lateral (**31**) and dorsal (**32**). Abbreviations: aedg. aedeagus, aec. aedeagal complex, aedg tn. aedeagal tine A2. anal vein, al. alula, bm. basal medial cell, br. basal radial cell, c. cerci, C. costa, CuA1,2, CuP. cubital veins, d. discal cell, db. dorsal bridge, DV. diagonal vein, e. epandrium, ej. ejaculatory apodeme, endaedg pr. endoaedeagal process, 1st fl. first flagellomere, gap. gonocoxal apodeme, gc. gonocoxite, gpr. gonocoxal process, gs. gonostylus, h. hypandrium, h. humeral cross vein, lat ej pr. lateral ejaculatory process, m3. third medial cell, pm. parameres, pm ap. parameral apodeme, pr. proctiger, pt. phallic plate, M1, M2, M3. medial veins, m-cu. cross vein between medial and cubital veins, pd, pedicel, R1, R2, R3+4, R5,Rs. radial veins, r-m. cross vein between redial and medial veins, Sc. subcostal vein, sc. Scape, st. stylus.

 Length: male body 10–16 mm, wing 9–15 mm. Female body 14–21 mm, wing 10–13 mm. Male: Frons shiny black with transverse white band; face rather short, snout-like, sides with grayish yellow pollinosity (Figs 23–25). Antenna blackish and pollinose (Fig. 26).

**Figures 33–41.**
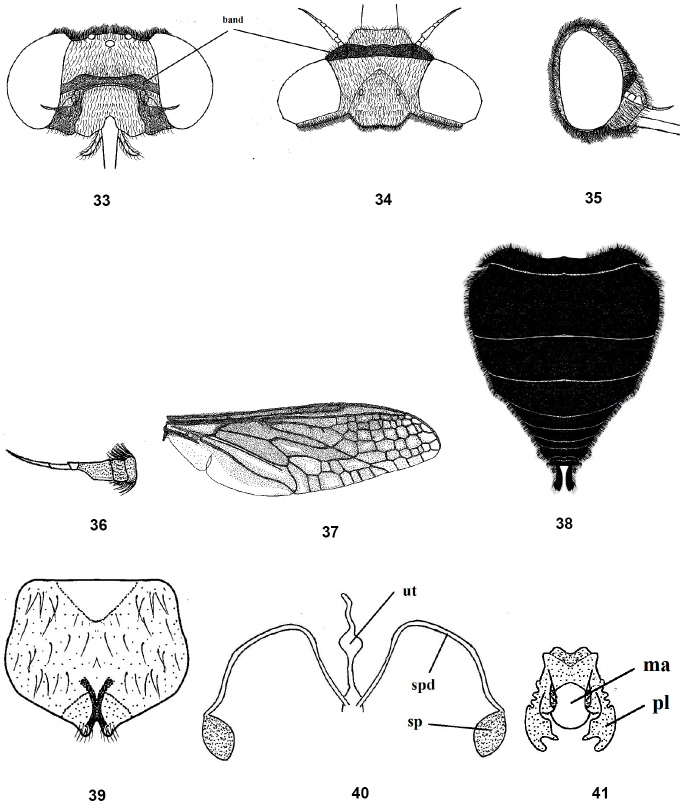
Female of *Nemestrinusater*, head, frontal (**33**), head, dorsal (**34**), head, lateral (**35**), antenna (**36**), wing (**37**), and abdomen (**38**). **39–41** female genitalia: subgenital plate (**39**), genital furca (**40**), and spermathecae (**41**). Abbreviations: ma. median aperture, pl. posterolateral projection, sp. spermatheca, spd. spermathecal duct, ut. uterus.

Thorax black or blackish brown, with blackish to yellowish brown hairs, pleurae with long and dense black hairs; leg blackish or dark yellow; claws well developed; pulvilli almost rudimentary. Wing blackish brown, but apex and postero-apical margin pale brown; wing with many small cells extend forward from R1 to hind margin (Fig. 27). Abdomen short, wide, reddish to orange with longitudinal black strip that is narrow posteriorly and sometimes absent at apex (Fig. 28). Male genitalia with gonocoxite having two processes, inner process short and slender, whereas the outer process is longer, thicker and subapically curved; gonostyli longer than the inner gonocoxal processes but shorter than the outer one, with subapical cleft and small projection (Figs 29, 30); aedeagus free, narrow distally and fused proximally with parameres; parameres slightly sinuate; parameral apodeme a long, while aedeagal tine is short; ejaculatory apodeme slender and narrow (Figs 31, 32). Female. Similar as male (Figs 33–37), except: eyes widely separated more than in male. Abdomen entirely black or at least with reddish black lateral margins (Fig. 38). Head in male slightly wider than thorax but in female narrower than thorax. Female genitalia with quadrate subgenital plate, bilobed distally (Fig. 39); genital furca with furcated arms and serrated laterally (Fig. 40); uterus small, with terminal accessory process; spermathecal ducts narrow and long with oval medium spermathecae (Fig. 41).

#### Local distribution.

 Coastal strip, Lower Nile.

#### Geographical distribution.

 Algeria, Egypt, Ethiopia, Israel, Spain, and Tunisia (Sack 1933; Bernardi 1973; Richter 1988).

#### Remarks.

*Nemestrinusabdominalis* and *N.fascifrons* are newly synonymized with *N.ater*. The earlier works of Lichtwardt (1909, 1919), Villeneuve (1912), and Bequaert (1938) suggested that *N.ater*, *N.abdominalis*, and *N.fascifrons* were closely related to each other based on Egyptian, Tunisian, and Palestinian material. We observed that *N.ater* has sexually dimorphic abdominal color. We also discovered that all the specimens previously identified by Efflatoun as *N.fascifrons* are males and we confirm these identifications. We also confirm that all the specimens that were previously identified by the same author as *N.ater* are females and confirmed by us as *N.fascifrons*. The two “species” of Efflatoun were captured from approximately the same locality and time of year by the same collector, i.e., “Efflatoun collected males at W. Garawi on 22.iii.1930 and 31.iii.1930 and females at W. Garawi on 22.iii.1930 and 31.iii.1930”; both are deposited in the Cairo University collection. We observed the sexual dimorphism and regard them as representing the same species.

### Nemestrinus exalbidus

Taxon classificationAnimaliaDipteraNemestrinidae

(Lichtwardt, 1907)

F362BA44-4E63-54EF-BC33-3B743528D59D

Figures 42–51 , 52–60

Nemestrina exalbidus Lichtwardt, 1907: 441. Type locality: Israel (Jerusalem).

#### Specimens examined.

W. Dar El-Maskhara 29°47'02.9"N, 31°24'59.9"E, 12.iv.1930 (1 f#); W. Hodein South Eastern Desert 23°5'14’"N, 35°19'45"E, 17.iii.1928 (1 m#); W. Hoff 29°53'02.6"N, 31°18'42.2"E, 12.iv.1921 (1 f#), 24.iii.1930 (1 m#); W. Zohleiga 26°07'59.9"N, 33°45'00.0"E, 27.iii.1925 (1 f#) (AZUC); Abu Rowash 30°04'30.7"N, 31°11'59.7"E, 16.iii.1927 (1 f#); Ogret El-Sheikh 29°52'50.1"N, 31°18'27.8"E, 31.iii.1926 (1 f#); W. Hoff 29°53'02.6"N, 31°18'42.2"E, 24.iii.1930 (1 m#); W. Rishrash 29°27'51"N, 31°22'2"E, 29.iii.1935 (7 m# & 4 f#) (CUC); Ogret El-Sheikh 29°52'50.1"N, 31°18'27.8"E, 14.iii.1927 (1 m#); W. Hoff 29°53'02.6"N, 31°18'42.2"E, 30.iii.1928 (1 m#); W. Zohleiga 26°07'59.9"N, 33°45'00.0"E, 25,29.iii.1925 (1 f#) (MAC); Etaka 29°26'19.1"N, 32°28'07.2"E , 22.ii.1951(1 f#), 26.iii.1951 (1 m#); Kerdasa 30°01'32.1"N, 31°06'27.5"E, 14.iv.1951 (3 f#); Mansouriah 29°58'05.3"N, 31°08'51.9"E, 25.iv.1957 (1 m#); W. Kaber 23°26'29"N, 25°50'23"E, 1.iv.1994 (1 f#) (ASUC).

#### Diagnosis.

 Frons covered with dense gray pollinosity except shiny black oval callus below ocellar triangle; wing hyaline, except slightly brownish along anterior margin, with a few small cells extending forward from R2+3 to M1 or M2. Abdomen orange or reddish with longitudinal median black vitta.

#### Redescription.

**Figures 42–51.**
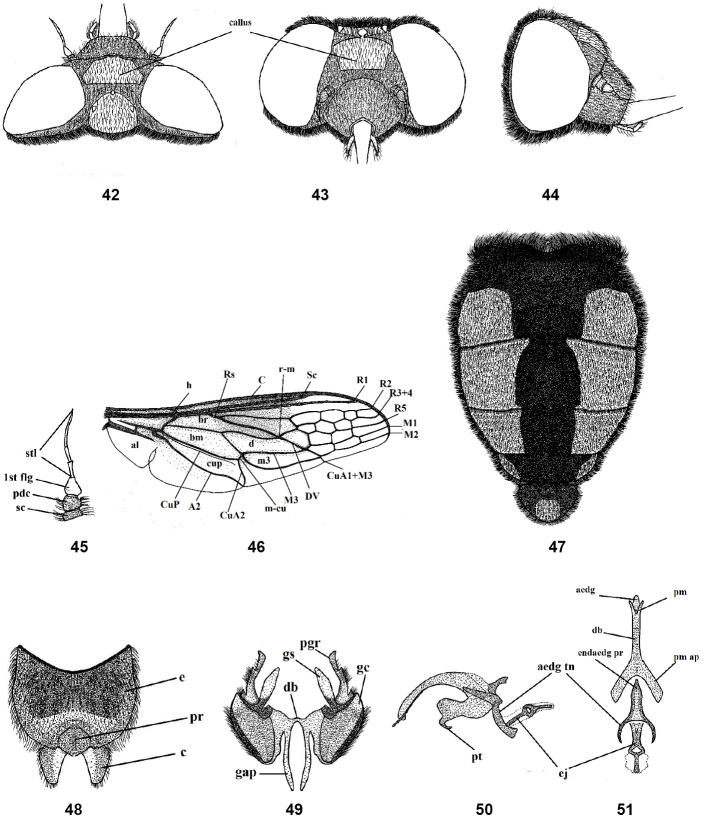
Male of *Nemestrinusexalbidus*, head, frontal (**42**), head, dorsal (**43**), head, lateral (**44**), antenna (**45**), wing (**46**), and abdomen (**47**). **48–51** male genitalia: epandrium, proctiger, and cerci (**48**), gonocoxite with gonostylus, ventral (**49**), aedeagal complex, lateral (**50**) and dorsal (**51**). Abbreviations: aedg. aedeagus, aec. aedeagal complex, aedg tn. aedeagal tine A2. anal vein, al. alula, bm. basal medial cell, br. basal radial cell, c. cerci, C. costa, CuA1,2, CuP. cubital veins, d. discal cell, db. dorsal bridge, DV. diagonal vein, e. epandrium, ej. ejaculatory apodeme, endaedg pr. endoaedeagal process, 1st fl. first flagellomere, gap. gonocoxal apodeme, gc. gonocoxite, gpr. gonocoxal process, gs. gonostylus, h. hypandrium, h. humeral cross vein, lat ej pr. lateral ejaculatory process, m3. third medial cell, pm. parameres, pm ap. parameral apodeme, pr. proctiger, pt. phallic plate, M1, M2, M3. medial veins, m-cu. cross vein between medial and cubital veins, pd, pedicel, R1, R2, R3+4, R5,Rs. radial veins, r-m. cross vein between redial and medial veins, Sc. subcostal vein, sc. Scape, st. stylus.

**Figures 52–60.**
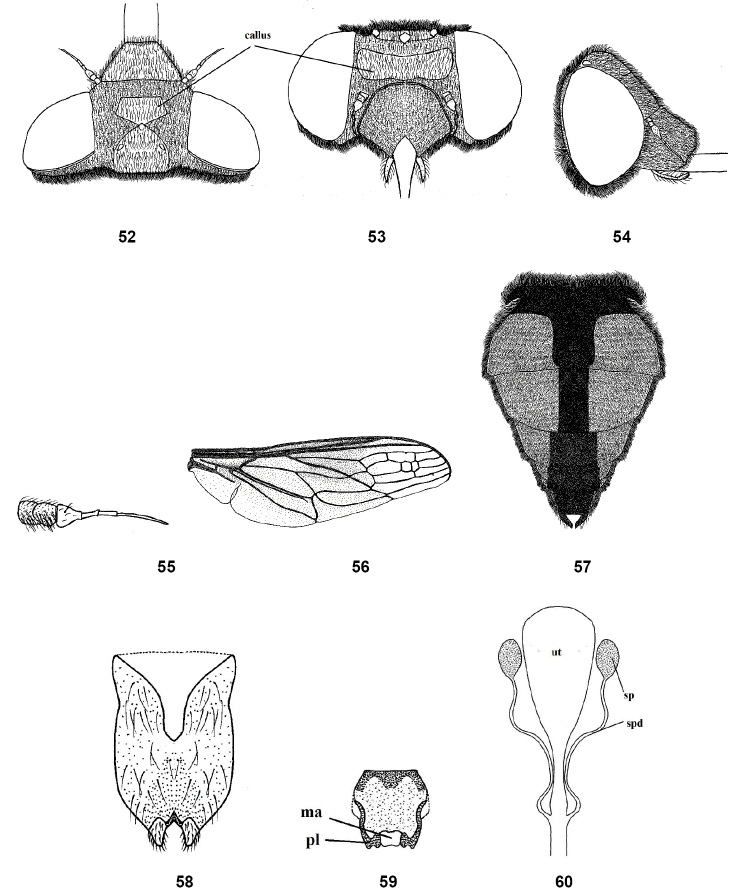
Female of *Nemestrinusexalbidus*, head, frontal (**52**), head, dorsal (**53**), head, lateral (**54**), antenna (**55**), wing (**56**), and abdomen (**57**). **58–60** female genitalia: subgenital plate (**58**), genital furca (**59**), and spermathecae (**60**). Abbreviations: ma. median aperture, pl. posterolateral projection, sp. spermatheca, spd. spermathecal duct, ut. uterus.

 Length: male body 14–17 mm, wing 12.5–14.5 mm. Female body 18 mm, wing 15.5 mm. Head shiny black with white hairs; frons covered with dense gray pollinosity except shiny black oval callus below ocellar triangle; face rather conical (Figs 42–44, 52–54). Thorax shiny black with dense whitish hairs laterally and a few dorsally. Leg orange, but femora black. Wing hyaline, except pale brownish anterior margin, with just a few small cells extending forward from R2+3 to M1 or M2 (Figs 46, 56). The differences in cell number and structure on the wing is continuous variation and inconsistently different between males and females. Abdomen orange or reddish with longitudinal black median vitta; base of abdomen covered with dense, short, yellowish gray hairs, lateral margins of subsequent segments with dense white hairs; venter of abdomen entirely orange (Figs 47, 57). Gonocoxite with only inner gonocoxal process, tapered apically (Figs 48, 49); distiphallus narrow; parameral apodeme rather short, aedeagal tine narrow and curved, forming semicircle, pointed distally; ejaculatory apodeme distally broader (Figs 50, 51). Female differentiated from male by the eyes that are more dichoptic. Female genitalia with rectangular subgenital plate, excavated proximally to approx. 1/2 length of plate (Fig. 58); genital furca with small genital aperture, between projections with broad ends, and curved posteromedially with small curve on upper and lower margins (Fig. 59); uterus large and flatted, spermathecae long (Fig. 60).

#### Local distribution.

 Eastern Desert, Lower Nile.

#### Geographical distribution.

 Egypt, Iran, and Israel (Sack 1933; Bernardi 1973; Richter 1988).

### Nemestrinus fasciatus

Taxon classificationAnimaliaDipteraNemestrinidae

(Olivier, 1811)

2EA43BCE-8DBE-5567-91C8-B55725DED1DE

Figures 61–70 , 71–79

Nemestrina fasciata Olivier, 1811: 171–172. Type locality: Egypt.

#### Specimens examined.

Burg El-Arab 30°54'12.7"N, 29°33'13.7"E, 6.v.1926 (1 m#); Burg El-Arab, 2.v.1921 (10 m#) (AZUC); Burg El-Arab 30°54'12.7"N, 29°33'13.7"E, 5.v.1926 (1 f#) (CUC); Burg El-Arab 30°54'12.7"N, 29°33'13.7"E, 10.v.1927 (1 m#), 19.iv.1928 (6 m# & 6 f#); King Mariut 30°57'27.2"N, 29°38'51.0"E 14.iv.1915 (1 m#), 23.v.1925 (1 m#); Burg El-Arab 30°54'12.7"N, 29°33'13.7"E, 2.v.1924 (1 f#) (MAC); Max 31°09'50.5"N, 29°51'47.7"E, 21.iv.1952 (1 f#) (ASUC).

#### Diagnosis.

 Frons and face with dense whitish hairs and pollinose; inner ends of transverse suture with two white spots; wing hyaline with brownish base, veins yellowish, without additional small cells; abdomen black with transverse white bands, slightly curved medially; gonocoxite with inner and outer processes, the inner tapered apically, outer slightly curved subapically; gonostyli broader than gonocoxal processes with broad subapical projection; aedeagal complex narrow, aedeagus slightly broader distally.

#### Redescription.

 Length: male body 13–16.5 mm, wing 1–12 mm. Female) body 12–19.5 mm, wing 1–13 mm.

**Figures 61–70.**
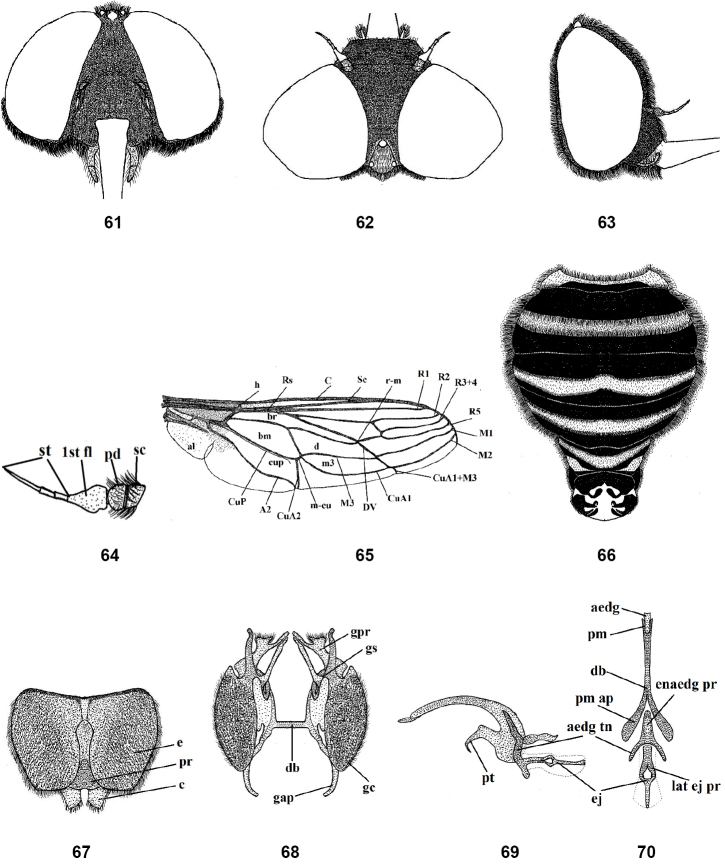
Male of *Nemestrinusfasciatus*, head, frontal (**61**), head, dorsal (**62**), head, lateral (**63**), antenna (**64**), wing (**65**), and abdomen (**66**). **67–70** male genitalia, epandrium, proctiger and cerci (**67**), gonocoxite with gonostylus, ventral (**68**), aedeagal complex, lateral (**69**) and dorsal (**70**). Abbreviations: aedg. aedeagus, aec. aedeagal complex, aedg tn. aedeagal tine A2. anal vein, al. alula, bm. basal medial cell, br. basal radial cell, c. cerci, C. costa, CuA1,2, CuP. cubital veins, d. discal cell, db. dorsal bridge, DV. diagonal vein, e. epandrium, ej. ejaculatory apodeme, enaedg pr. endoaedeagal process, 1st fl. first flagellomere, gap. gonocoxal apodeme, gc. gonocoxite, gpr. gonocoxal process, gs. gonostylus, h. hypandrium, h. humeral cross vein, lat ej pr. lateral ejaculatory process, m3. third medial cell, pm. parameres, pm ap. parameral apodeme, pr. proctiger, pt. phallic plate, M1, M2, M3. medial veins, m-cu. cross vein between medial and cubital veins, pd, pedicel, R1, R2, R3+4, R5,Rs. radial veins, r-m. cross vein between redial and medial veins, Sc. subcostal vein, sc. Scape, st. stylus.

**Figures 71–79.**
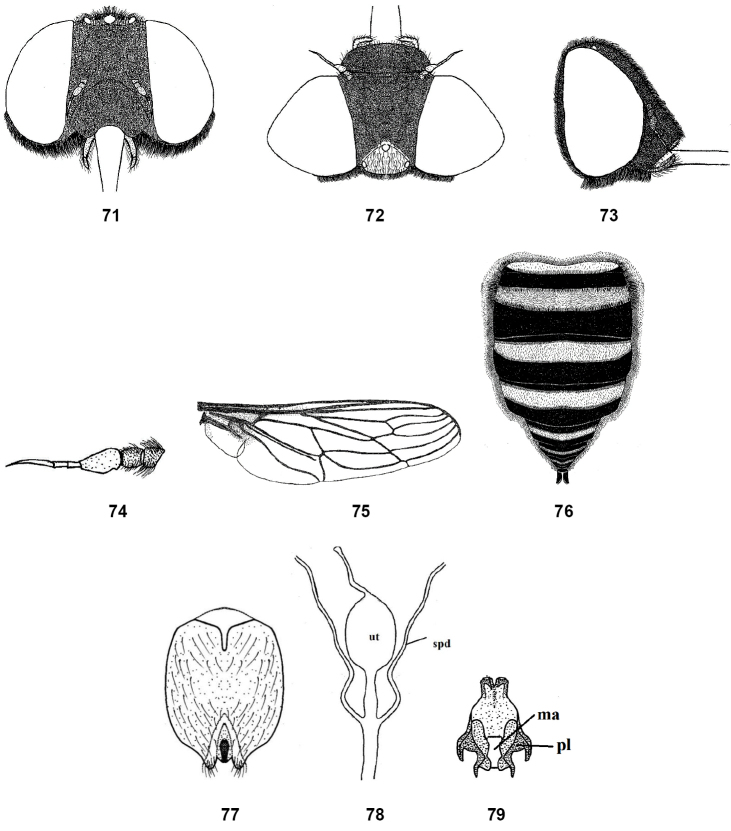
Female of *Nemestrinusfasiciatus*, head, frontal (**71**), head, dorsal (**72**), head, lateral (**73**), antenna (**74**), wing (**75**), and abdomen (**76**). **77–79** female genitalia: subgenital plate (**77**), genital furca (**78**), and spermathecae (**79**). Abbreviations: ma. median aperture, pl. posterolateral projection, sp. spermatheca, spd. spermathecal duct, ut. uterus.

Head triangular in profile, ventrally with dense, short, whitish hairs; frons and face with dense whitish hairs and pollinosity (Figs 61–63, 71–73); antenna distinctly jointed, stylus is brown (Figs 64, 74). Thorax slightly shiny black; inner parts of transverse suture with two white spots; scutellum and mesonotum with grayish yellow hairs; pleurae with long white hairs. Leg with blackish femora covered with whitish hairs; tibiae and tarsi brown with brownish red hairs; pulvilli orange, nearly as long as claws. Wing hyaline with brownish infuscate base; veins yellowish, without additional small cells (Figs 65, 75). Abdomen black with transverse white bands, whi slightly curved medially; basal segments with long yellowish hairs but subsequent segments with white hairs; venter of abdomen with dense white hairs that fold on the lateral margins (Figs 66, 76). Gonocoxite with inner and outer processes, inner tapered apically, outer slightly curved subapically; gonostyli broader than gonocoxal processes with broad projection subapically (Figs 67, 68); aedeagal complex narrow, aedeagus slightly broader apically (Figs 69, 70).

Female: eyes separated in both sexes but considerably broader than in male at vertex; genitalia with sub-rectangular subgenital plate (Fig. 77); genital furca narrower anteriorly with four incurved posterolateral projections (Fig. 78); uterus with terminal accessory process; spermathecae rather long (Fig. 79).

#### Local distribution.

 Coastal strip.

#### Geographical distribution.

 Algeria, Egypt, Morocco, Israel, and Syria (Sack 1933; Bernardi 1973; Richter 1988).

### Nemestrinus reticulatus

Taxon classificationAnimaliaDipteraNemestrinidae

Latreille, 1802

12D5534B-BDDB-5096-ABBF-E58113D7A687

Figures 80–85

Nemestrinus reticulatus Latreille, 1802: 437. Type locality: not given but according to Latreille (1809: 307), it is Egypt and Syria.
Rhynchocephalus latreillei Fischer, 1812: 195.
Nemestrina cinctus Macquart, 1840: 16.
Nemestrina kindermanni Bischof, 1905: 172.

#### Diagnosis.

Frons with shiny yellow or black spot below ocelli; mesonotum with two gray spots at inner ends of the transverse suture, between them there is a thin longitudinal stripe; wing hyaline in posterior 1/2 and apex but brownish on anterior 1/2 and slightly infuscate at base; wing with small cells that extend forward from R_2_ to hind margin; abdomen gray, matte, with incomplete transverse black stripes; tergite II bears shiny black spots divided in the middle by a transverse longitudinal gray strip; black spots on tergites III–V more or less fused into bands with an emargination along the posterior margin; on tergite III, gray emargination varies from very deep to nearly absent; abdominal venter with dense gray pollinosity, the second sternite with central black spot.

#### Description.

**Figures 80–85.**
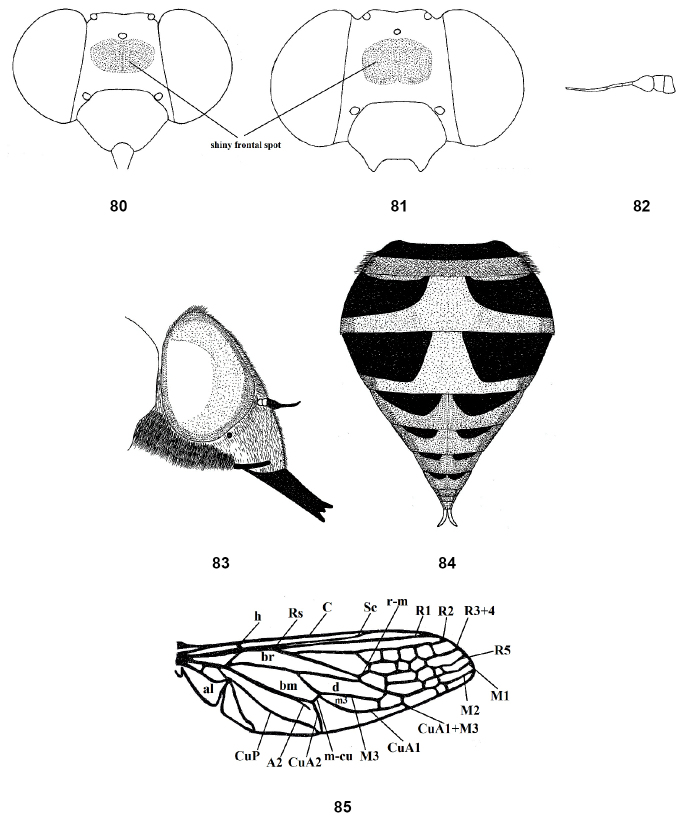
*Nemestrinusreticulatus*, male head, frontal (**80**), female head, frontal (**81**), male antenna (after Bequaert, 1938) (**82**), male head, lateral (**83**), female abdomen (after Sack 1933) (**84**), and wing (after Seguy, 1926) (**85**). Abbreviations: A2. anal vein, al. alula, bm. basal medial cell, br. basal radial cell, C. costa, CuA1,2, CuP. cubital veins, d. discal cell, DV. diagonal vein, h. humeral cross vein, m3. third medial cell, M1, M2, M3. medial veins, m-cu. cross vein between medial and cubital veins, R1, R2, R3+4, R5,Rs. radial veins, r-m. cross vein between redial and medial veins, Sc. subcostal vein.

 Length: body 14–15 mm. Head black with dense gray pollinosity and whitish hairs; frons with shiny yellow or black spot below ocelli, in male frons at vertex nearly as wide as eye width (Figs 80, 83), while in female nearly twice as eye width (Fig. 81); antenna with orange scape and pedicel, first flagellomere brown to blackish brown with some gray pollinosity (Fig. 82), basal two segments of stylus subequal in length and segment III 2/3 × longitudinal eye diameter; palpi yellow or brown with black apices. Thorax pale black with yellowish white or grayish white hairs, but longer and denser on scutellum and pleurae; mesonotum with two gray spots at inner ends of transverse suture and between them is a thin longitudinal stripe. Leg rusty red; in females, only hind tarsi blackish or hind leg entirely blackish; in males, all femora black; hind tibiae and tarsi blackish. Wing hyaline over posterior 1/2 and at apex, but somewhat brown over anterior 1/2 and slightly infuscate at base; wing with small cells that extend forward from R_2_ to hind margin (Fig. 85). Abdomen gray, matte, with incomplete transverse black stripes; tergite II with shiny black spots divided in the middle by a transverse longitudinal gray strip; black spots on tergites III–V are more or less fused into bands with an emargination along the posterior margin; on tergite III, gray emargination varies from very deep to nearly absent; abdominal venter with dense gray pollinosity, sternite II with black central spot (Fig. 84).

#### Local distribution.

 Unknown.

#### Geographical distribution.

 Armenia, Egypt, Greece, Russia (Caucasus), Saudi Arabia, Syria, and Turkey (Sack 1933; Bernardi 1973).

#### Remarks.

 This species is not represented in Egyptian collections nor in the field. We include the species here and in the key below as it has been recorded from Egypt (Sack 1933 & Bernardi 1973 & Richter 1988); future research might reveal its presence in this part of Africa.

### Nemestrinus rufipes

Taxon classificationAnimaliaDipteraNemestrinidae

(Olivier, 1811)

BA0266A8-0336-5757-B4F4-871B85C75DC9

Figures 1 , 2 , 86–95 , 96–104

Nemestrina rufipes Olivier, 1811: 171.
Nemestrina lateralis Wiedemann, 1828: 560.
Nemestrina ruficornis Macquart, 1840: 15. Syn. nov.

#### Type material.

*Nemestrinusrufipes*: Type male, “Aegypten”, Egypt (ZMHB) (pers. comm. Mr. Sven Marotzke). *Nemestrinalateralis*: Type female, “Aegypten”, Egypt (ZMHB) (pers. comm. Mr. Sven Marotzke).

#### Specimens examined.

 Ezbet El-Nakhl 30°08'22.6"N, 31°19'27.8"E, 20.iv.1921 (1 m# & 3 f#); Helwan 29°50'37.6"N, 31°19'05.0"E, 8.iv.1932 (2 f#); W. Morrah 22°22'39.1"N, 33°46'00.3"E, 26.iii.1927 (1 m#) (AZUC); Abu Rawash 30°04'30.7"N, 31°11'59.7"E, 13.iii.1955 (3 m# & 4 f#), 17.iii.1955 (1 m#); Kerdasa 30°01'32.1"N, 31°06'27.5"E, 11.iv.1926 (1 m#); Giza 30°00'40.0"N, 31°11'31.4"E, 7.v.1955 (1 m#), 2.iii.1927 (1 m#); Helwan 29°50'37.6"N, 31°19'05.0"E, 8.iv.1932 (2 f#), 3.iv.1934 (1 m# & 3 f#), 8.iv.1934 (6 f#), 17.iii.1934 (1 f#), 17.iv.1934 (1 f#), 23.iv.1935 (3 f#); W. Garawi 29°47'43.9"N, 31°25'54.9"E, 31.iii.1930 (1 f#) (CUC); Abu Rawash 30°04'30.7"N, 31°11'59.7"E, 11.iv.1925 (1 m#), 17.iv.1925 (2 m# & 1 f#), 3.iv.1926 (1 m# & 1 f#),12.iii.1936 (1 f#), 4.iv.1961 (3 f#); (Noaman Bey) Alexandria 31°10'03.5"N, 29°51'56.2"E, (1 f#, without date); Assyut 27°23'00.0"N, 31°44'38.0"E, 2.iv.1917 (1 f#); Bent Suef 29°04'N, 31°05'E, iii.1965 (1 m#); Dakhla Mout 25°32'41.4"N, 28°55'44.0"E, 17.iii.1934 (1 m#); El-Mallah 30°00'37.7"N, 31°09'34.0"E , 14.v.1927 (1 m#); Gabal El-Halal 30°39'10.8"N, 34°01'43.9"E, 25.iv.1924 (1 m# & 1 f#); Gabal El-Sanadiq, 5.iv.1934 (1 f#); 12.iv.1924 (1 m#); Kafr Hakim 30°04'39.7"N, 31°06'46.3"E, 7.iv.1924 (1 m#), 14.iv.1925 (1 m#), 20.iv.1925 (1 m# & 1 f#); Mansouriah 29°58'05.3"N, 31°08'51.9"E, 28.iv.1926 (1 f#), 4.iii.1934 (1 m#); Marg, 1.iv.1923 (1 f#); Marsa Matrouh 31°11'04.1"N, 27°15'42.4"E, 19.i.1933 (1 m#), 17.iii.1933 (1 m#), 1.iv.1961 (1 f#); W. Garawi 29°47'43.9"N, 31°25'54.9"E, 3.iii.1925 (1 f#); W. Um Elek 29°52'59.9"N, 31°31'00.1"E, 28.iii.1918 (1 f#); W. Zohleiga 26°07'59.9"N, 33°45'00.0"E, 25–29.iii.1925 (1 f#) (MAC); W. Digla 29°59'00.1"N, 31°19'41.2"E, 5.iv.1952 (1 m#) (ASUC). Aegypten (1 m# & 1f#), without date (ZMHB) sent by Mr. Sven Marotzke and Bernhard Schurian; Dakahlia, Mansuriya 31.1656° N, 31.4913°E 31.iii.1964 (1 m# & 1 f#) specimen numbers: male: USNMENT01371563, female: USNMENT01371564, Cairo, Marg 30.1543°N, 31.3484°E (1 f#), without date, specimen number USNMENT01371561 (USNM) (identified by Dr. Torsten Dikow).

#### Diagnosis.

 Frons covered with dense orange yellow pollinosity except with shiny blackish brown transverse oval callus below dark orange ocellar triangle; wing with yellowish brown band in the middle, but clear in apical part and along posterior margin; wing with small cells extending forward from R2 to M1 or M2. Abdomen orange to reddish orange with longitudinal black median vitta; first tergite entirely black, tergite II on anterior margin with transverse white band. Gonocoxite with inner process slightly tapered; gonostyli longer than gonocoxal process, curved subapically with small projection; aedeagus fused proximally with parameres and separated distally, parameres and aedeagus with small indentations distally in lateral view.

#### Redescription.

**Figures 86–95.**
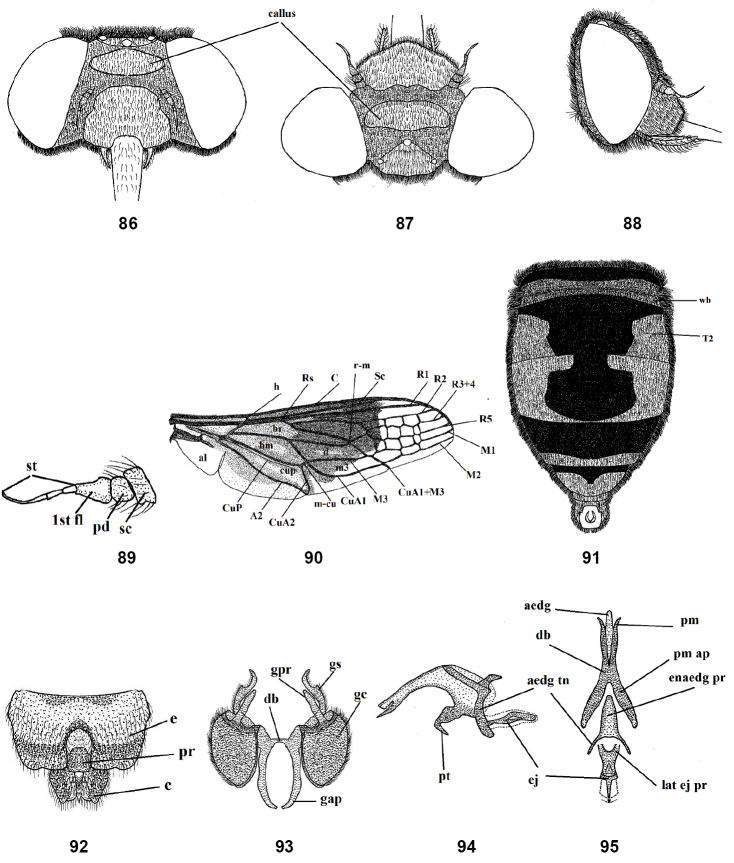
Male of *Nemestrinusrufipes*, head, frontal (**86**), head, dorsal (**87**), head, lateral (**88**), antenna (**89**), wing (**90**), and abdomen (**91**). **92–95** male genitalia: epandrium, proctiger and cerci (**92**), gonocoxite with gonostylus, ventral (**93**), aedeagal complex, lateral (**94**) and dorsal (**95**). Abbreviations: aedg. aedeagus, aec. aedeagal complex, aedg tn. aedeagal tine A2. anal vein, al. alula, bm. basal medial cell, br. basal radial cell, c. cerci, C. costa, CuA1,2, CuP. cubital veins, d. discal cell, db. dorsal bridge, DV. diagonal vein, e. epandrium, ej. ejaculatory apodeme, enaedg pr. endoaedeagal process, 1st fl. first flagellomere, gap. gonocoxal apodeme, gc. gonocoxite, gpr. gonocoxal process, gs. gonostylus, h. hypandrium, h. humeral cross vein, lat ej pr. lateral ejaculatory process, m3. third medial cell, pm. parameres, pm ap. parameral apodeme, pr. proctiger, pt. phallic plate, M1, M2, M3. medial veins, m-cu. cross vein between medial and cubital veins, pd, pedicel, R1, R2, R3+4, R5,Rs. radial veins, r-m. cross vein between redial and medial veins, Sc. subcostal vein, sc. Scape, st. stylus, T2. tergite 2, wb. White band.

**Figures 96–104.**
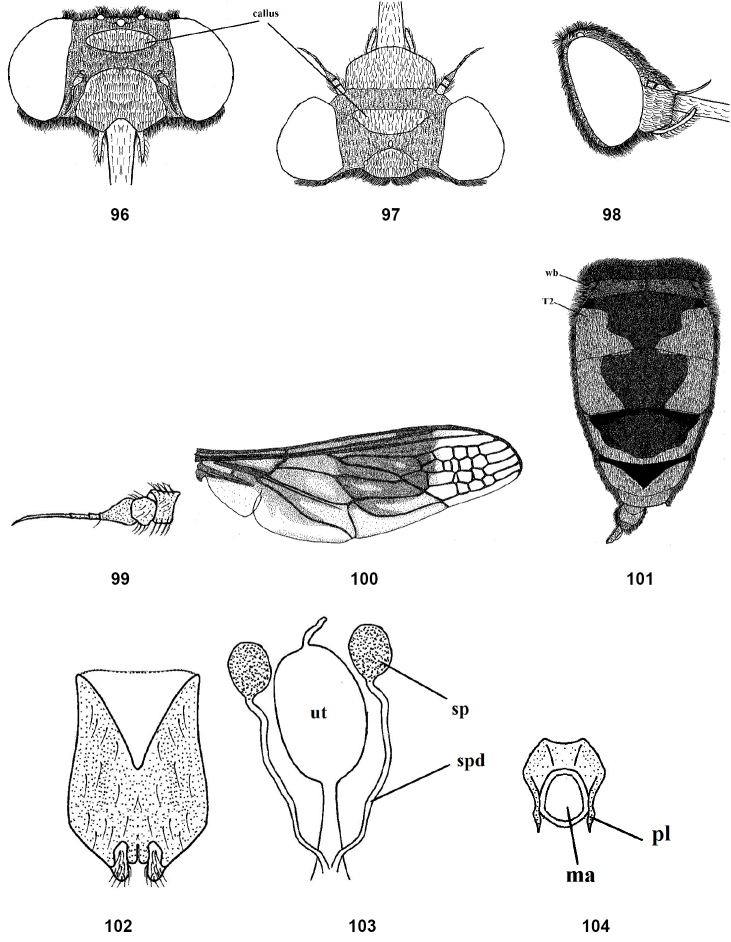
Female of *Nemestrinusrufipes*, head, frontal (**96**), head, dorsal (**97**), head, lateral (**98**), antenna (**99**), wing (**100**) and abdomen (**101**). **102–104** female genitalia: subgenital plate (**102**), genital furca (**103**), and spermathecae (**104**). Abbreviations: ma. median aperture, pl. posterolateral projection, sp. spermatheca, spd. spermathecal duct, ut. uterus, T2. tergite 2, wb. White band.

 Length: male body13.5–18.5 mm, wing 11.5–16 mm. Female body 14–21 mm, wing 12–17.5 mm. Head short, wider than thorax; frons covered with dense orange-yellow pollinosity except with shiny blackish brown transverse oval callus (Figs 86–88, 96–98); face shiny brownish orange with short yellow hairs; antenna orange (Figs 89, 99); proboscis black, as long as thorax, upper surface of base with short yellow hairs; palpi orange. Thorax shiny black with yellow hairs, longer and denser on the sides and in front; mesonotum with indistinct spots at inner ends of transverse suture. Leg orange, coxae and base of femora somewhat brown, pulvilli light yellow and nearly 1/2 length of claw. Wing with yellowish brown band in the middle, but clear apically and along posterior margin; wing with small cells extending forward from R2 to M1 or M2 (Figs 90, 100). Halter brown with light yellow pedicel. Abdomen orange to reddish orange with longitudinal black median vitta; tergite I entirely black, tergite II with transverse white band; abdomen with short and golden yellow hairs but longer laterally; abdominal venter orange and with black lateral margins (Figs 91, 101). Gonocoxite with inner process slightly tapered; gonostyli longer than gonocoxal process, curved subapically with small projection (Fig. 93); aedeagus fused proximally with parameres and separated distally, parameres and aedeagus with small indentations distally in lateral view (Figs 94, 95). Female genitalia: rectangular subgenital plate with large curve (Fig. 102); genital furca with large aperture surrounded by narrow and slightly curved posterolaterally projections (Fig. 103); uterus with small terminal accessory; spermathecae nearly as long as the uterus (Fig. 104).

#### Local distribution.

 Coastal strip, Lower Nile, Upper Nile, Sinai.

#### Geographical distribution.

 Algeria, Egypt, and Syria (Sack 1933; Bernardi 1973; Richter 1988).

#### Remarks.

Bernardi (1973) and Richter (1988) considered *N.rufipes* and *N.ruficornis* to be valid species but, in contrast, Lichtwardt (1909) and Bequaert (1938) synonymized the two. We agree with this decision based on a comparison of the original description of *N.ruficornis* (no material was examined) with the male type specimen of *N.rufipes* (Fig. 2) and the female type specimen of *N.lateralis* (Fig. 3), both in ZMHB, in addition to both sexes of many old Egyptian specimens of *N.rufipes*. Thus, we confirm that *N.rufipes* and *N.ruficornis* are conspecific and the first is the valid name, and *Nemestrinusruficornis* is here synonymized with *N.rufipes*.

#### List of doubtful species.

In the present study, three species are treated as doubtful and are excluded from the list of Egyptian Nemestrinidae: *Nemestrinuscaucasicus*, *Nemestrinuspallipes*, and *Nemestrinuspersicus*.

## Discussion

As a result of this revision, we can confirm six species of *Nemestrinus* present in Egypt. This is lower than the 12 taxa (eleven species and one subspecies) listed by Steyskal and El-Bialy (1967) but three species are treated as doubtfully occurring including *N.pallipes* added by Bernardi (1973) and Richter (1988) as Egyptian species and three species that are newly synonymized (*N.abdominalis*, *N.fascifrons*, and *N.ruficornis*). Furthermore, *N.jullieni*, a subspecies designated by Steyskal and El-Bialy (1967), is confirmed as a synonym of *N.aegyptiacus*.

The first species (*N.caucasicus*) does not occur in Egypt according to Sack (1933), Bequaert (1938), Bernardi (1973), Richter (1988), Narchuk (2007), or Kocak and Kemal (2013), whereas Paramonov (1951) reported it from North Africa but without examining any Egyptian material. It is also listed by Steyskal and El-Bialy (1967) citing the literature but not represented in the Egyptian reference collections.

The second species (*N.pallipes*) is not represented in Egyptian collections. This species was previously considered to be an Egyptian species based on an erroneous interpretation of its type locality (Java) by Bequaert (1932), Bernardi (1973), and Richter (1988), for reasons unknown. The type locality was given by Olivier (1811) in his original description as Java, while describing seven new species from Egypt, although two of them were from Arabia and around the Caspian Sea. Olivier (1811) based his paper on material collected from different areas in the Middle East and his “Java” evidently refers to a place near Tel-Aviv in Israel currently known as Jaffa, not an Egyptian locality. We communicated with managers in Tel Aviv museum (Steinhardt museum), Diptera collection (Dr. Elizabeth Morguilis and Dr. Ariel-Leib Frieman), who checked the nemestrinid group and they do not have any specimens of this species in their collections. This species is also not mentioned in the list of Steyskal and El-Bialy (1967). Although it is believed that this species does not occur in Egypt, it may yet be found and recorded from the country.

The third species (*N.persicus*) is reported in Sack (1933) and Paramonov (1945) as an Egyptian species, but without any listing of Egyptian material. It is also mentioned as an Egyptian species by Steyskal and El-Bialy (1967) but is not represented in Egyptian reference collections; however, Bequaert (1932), Bernardi (1973), and Richter (1988) excluded it as an Egyptian species. The type locality in the original description was given as Iran by Lichtwardt (1909) and, consequently, this species is excluded from the Egyptian fauna.

*Nemestrinusreticulatus* is stated here as not having any specimens in Egyptian collections and is not excluded from the Egyptian fauna in our study because we trust the descriptions of Latreille (1809) who originally reported the species in Egypt. Our drawings of this species are reproduced from Bequaert (1938), Sack (1933), and Seguy (1926).

**Map 1.**
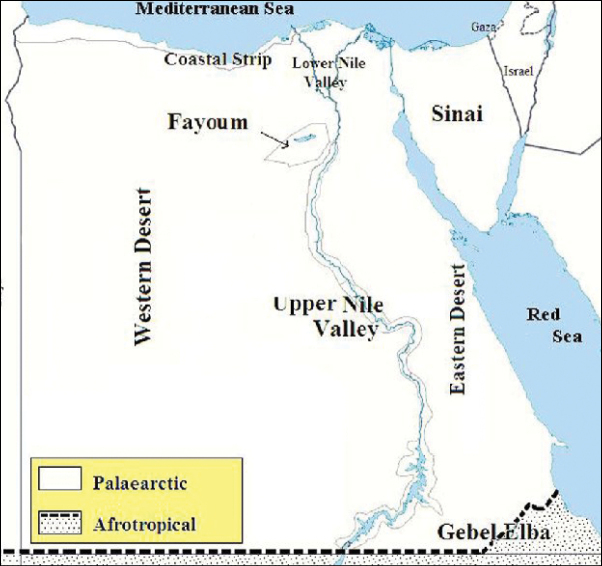
Map of Egypt showing the ecological zones (after elhawagry and gilbert 2014).

As we observed on the maps there are similarities in the distributions of *N.aegyptiacus* and *N.rufipes*, which are longitudinally scattered from Lower to Upper Egypt and the western and eastern deserts, while *N.faciatus* is concentrated only in some localities on the coastal strip in Alexandria. *Nemestrinusexalbidus* is dispersed around the lower Egyptian delta and a few localities in the western and eastern deserts. *Nemestrinusater* has a crosswise distribution in the northern area of Egypt including Sinai, and the lower and upper Nile valley (see Map 1), and one record in the eastern desert, in addition to one locality near Libya.

**Map 2.**
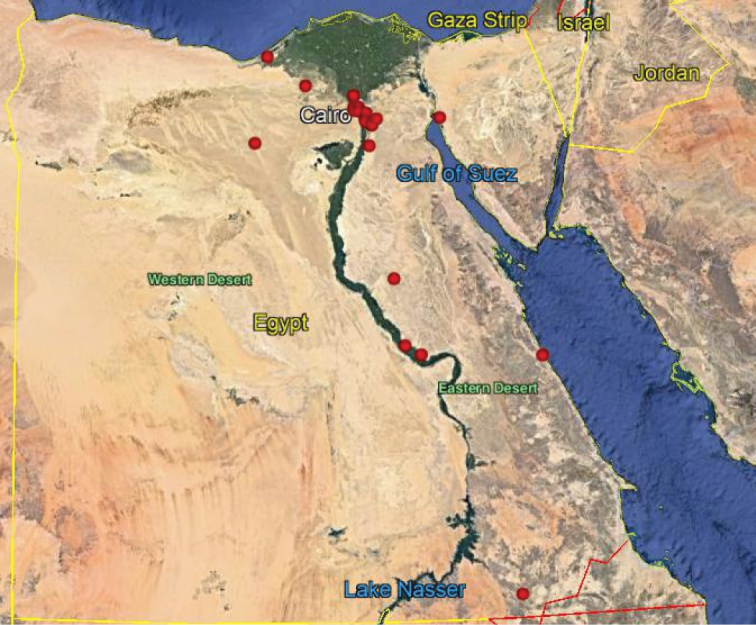
Distribution of the species *N.aegyptiacus* in Egypt.

**Map 3.**
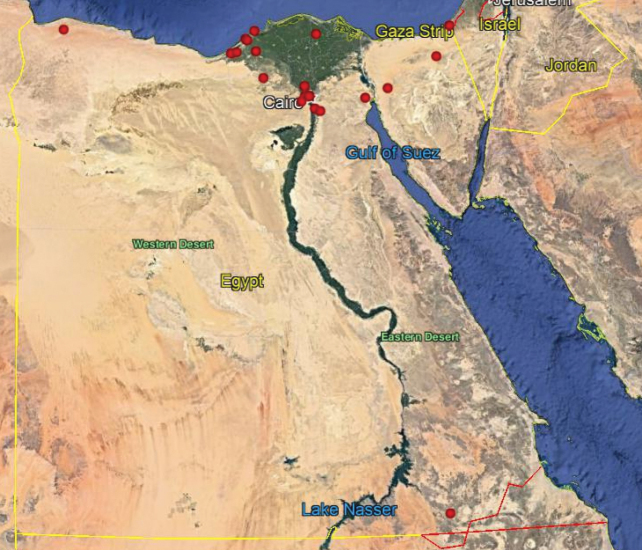
Distribution of the species *N.ater* in Egypt.

**Map 4.**
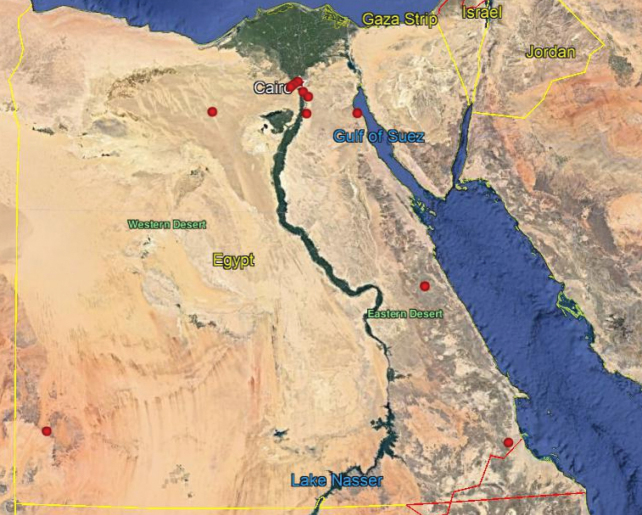
Distribution of the species *N.exalbidus* in Egypt.

**Map 5.**
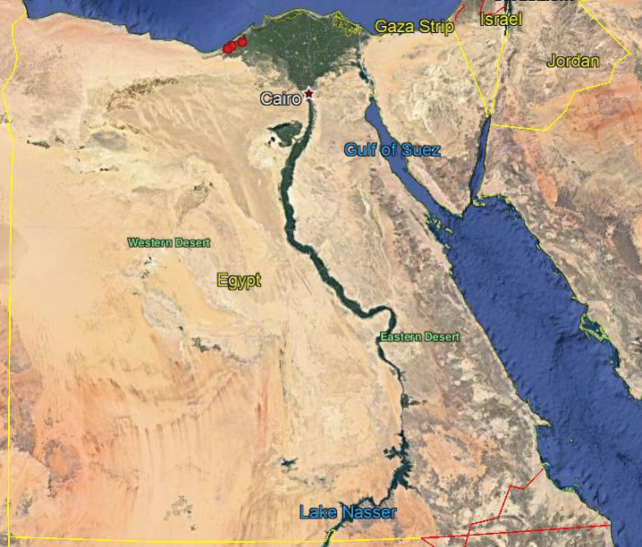
Distribution of the species *N.fasciatus* in Egypt.

**Map 6.**
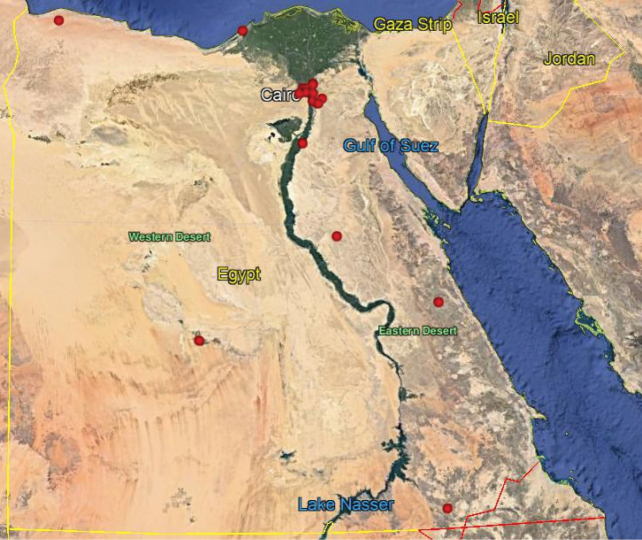
Distribution of the species *N.rufipes* in Egypt.

The species of *Nemestrinus* are concentrated in the semiarid areas around the Nile delta, especially around Lower Egypt and in some arid areas in western, eastern, and the Egyptian Sinai deserts. The wider geographical distribution of *Nemestrinus* in the adjacent countries includes North Africa (Algeria, Libya, Morocco, and Tunisia) which all have a large percentage of the arid deserts that these nemestrinid species prefer. And at nearly the same latitude are Israel, Saudi Arabia, and Syria which also have arid areas (deserts) and may support species.

We found based that the seasonal imago flight activity of all *Nemestrinus* species in Egypt is in the spring season (March, April, and May) and only the species *N.ater* and *N.exalbidus* may also be activate in February.

## Supplementary Material

XML Treatment for Nemestrinus

XML Treatment for Nemestrinus aegyptiacus

XML Treatment for Nemestrinus ater

XML Treatment for Nemestrinus exalbidus

XML Treatment for Nemestrinus fasciatus

XML Treatment for Nemestrinus reticulatus

XML Treatment for Nemestrinus rufipes
